# Supplementary material for: Combining antibiotics with antivirulence compounds can have synergistic effects and reverse selection for antibiotic resistance in Pseudomonas aeruginosa
Source: PLoS Biol. 2020 Aug 18;18(8):e3000805. doi: 10.1371/journal.pbio.3000805 (PMC7433856; doi:10.1371/journal.pbio.3000805)
Supplement: S1 Table — (DOCX) [file pbio.3000805.s011.docx]

| **Combination** | **Antibiotic presence** | **Factor^2^** | **Single comparisons^1,2^** | **Degrees of**  **freedom^1,2^** | **F value^2^** | **t value^1,2^** | **p-value^1,2^** |
| --- | --- | --- | --- | --- | --- | --- | --- |
| Ciprofloxacin-Gallium |  |  |  |  |  |  |  |
|  | - Atb |  | 0 vs reference line | 15 |  | -5.71 | 4.121 x 10^-5^ |
|  |  |  | Low vs reference line | 7 |  | -13.56 | 2.786 x 10^-6^ |
|  |  |  | Intermediate vs reference line | 7 |  | 2.51 | 0.040 |
|  | + Atb |  | 0 vs reference line | 15 |  | 7.27 | 2.749 x 10^-6-^ |
|  |  |  | Low vs reference line | 15 |  | 2.54 | 0.022 |
|  |  |  | Intermediate vs reference line | 15 |  | 2.17 | 0.046 |
|  |  | Treatment |  | 2 | 4.17 |  | 0.022 |
|  |  | Residuals |  | 45 |  |  |  |
|  |  |  | 0 vs Low |  |  | -1.82 | 0.076 |
|  |  |  | 0 vs Intermediate |  |  | -2.85 | 0.007 |
| Colistin-Gallium |  |  |  |  |  |  |  |
|  | - Atb |  | 0 vs reference line | 15 |  | -13.50 | 8.518 x 10^-10^ |
|  |  |  | Low vs reference line | 7 |  | 8.86 | 4.718 x 10^-5^ |
|  |  |  | Intermediate vs reference line | 7 |  | 3.16 | 0.016 |
|  | + Atb |  | 0 vs reference line | 15 |  | 13.12 | 1.266 x 10^-9^ |
|  |  |  | Low vs reference line | 15 |  | 17.15 | 2.901 x 10^-11^ |
|  |  |  | Intermediate vs reference line | 15 |  | 7.02 | 4.139 x 10^-6^ |
|  |  | Treatment |  | 2 | 15.31 |  | 8.482 x 10^-6^ |
|  |  | Residuals |  | 45 |  |  |  |
|  |  |  | 0 vs Low |  |  | 5.49 | 1.760 x 10^-6^ |
|  |  |  | 0 vs Intermediate |  |  | 2.16 | 0.036 |
| Meropenem-Gallium |  |  |  |  |  |  |  |
|  | - Atb |  | 0 vs reference line | 15 |  | -6.20 | 1.691 x 10^-5^ |
|  |  |  | Low vs reference line | 7 |  | -1.87 | 0.104 |
|  |  |  | Intermediate vs reference line | 7 |  | -4.48 | 0.003 |
|  | + Atb |  | 0 vs reference line | 15 |  | 5.80 | 3.480 x 10^-5^ |
|  |  |  | Low vs reference line | 15 |  | 4.65 | 3.140 x 10^-4^ |
|  |  |  | Intermediate vs reference line | 15 |  | 0.72 | 0.482 |
|  |  | Treatment |  | 2 | 12.30 |  | 5.495 x 10^-5^ |
|  |  | Residuals |  | 45 |  |  |  |
|  |  |  | 0 vs Low |  |  | -0.87 | 0.390 |
|  |  |  | 0 vs Intermediate |  |  | -4.66 | 2.810 x 10^-5^ |
| Tobramycin-Gallium |  |  |  |  |  |  |  |
|  | - Atb |  | 0 vs reference line | 19 |  | -6.30 | 4.768 x 10^-6^ |
|  |  |  | Low vs reference line | 7 |  | -2.49 | 0.042 |
|  |  |  | Intermediate vs reference line | 7 |  | -3.90 | 0.006 |
|  | + Atb |  | 0 vs reference line | 19 |  | 8.42 | 7.763 x 10^-8^ |
|  |  |  | Low vs reference line | 19 |  | -0.16 | 0.877 |
|  |  |  | Intermediate vs reference line | 19 |  | 5.71 | 1.675 x 10^-5^ |
|  |  | Treatment |  | 2 | 22.99 |  | 4.766 x 10^-8^ |
|  |  | Residuals |  | 57 |  |  |  |
|  |  |  | 0 vs Low |  |  | -6.78 | 7.360 x 10^-9^ |
|  |  |  | 0 vs Intermediate |  |  | -3.38 | 0.001 |
| Ciprofloxacin-Furanone C30 |  |  |  |  |  |  |  |
|  | - Atb |  | 0 vs reference line | 15 |  | -12.56 | 2.312 x 10^-9^ |
|  |  |  | Low vs reference line | 7 |  | -10.50 | 1.546 x 10^-5^ |
|  |  |  | Intermediate vs reference line | 7 |  | -0.22 | 0.835 |
|  | + Atb |  | 0 vs reference line | 15 |  | 13.41 | 9.315 x 10^-10^ |
|  |  |  | Low vs reference line | 15 |  | 35.13 | 8.04 x 10^-16^ |
|  |  |  | Intermediate vs reference line | 15 |  | 51.13 | < 2.2 x 10^-16^ |
|  |  | Treatment |  | 2 | 3.61 |  | 0.035 |
|  |  | Residuals |  | 45 |  |  |  |
|  |  |  | 0 vs Low |  |  | 0.44 | 0.659 |
|  |  |  | 0 vs Intermediate |  |  | -2.07 | 0.044 |
| Colistin-Furanone C30 |  |  |  |  |  |  |  |
|  | - Atb |  | 0 vs reference line | 19 |  | -3.38 | 0.003 |
|  |  |  | Low vs reference line | 15 |  | -3.85 | 0.001 |
|  |  |  | Intermediate vs reference line | 15 |  | -3.20 | 0.006 |
|  | + Atb |  | 0 vs reference line | 19 |  | 4.54 | 2.223 x 10^-4^ |
|  |  |  | Low vs reference line | 19 |  | 5.30 | 4.121 x 10^-5^ |
|  |  |  | Intermediate vs reference line | 19 |  | 2.48 | 0.023 |
|  |  | Treatment |  | 2 | 13.73 |  | 1.358 x 10^-5^ |
|  |  | Residuals |  | 57 |  |  |  |
|  |  |  | 0 vs Low |  |  | -4.32 | 6.320 x 10^-5^ |
|  |  |  | 0 vs Intermediate |  |  | -4.73 | 1.530 x 10^-5^ |
| Meropenem-Furanone C30 |  |  |  |  |  |  |  |
|  | - Atb |  | 0 vs reference line | 15 |  | -2.62 | 0.019 |
|  |  |  | Low vs reference line | 7 |  | -1.57 | 0.159 |
|  |  |  | Intermediate vs reference line | 7 |  | -1.70 | 0.133 |
|  | + Atb |  | 0 vs reference line | 15 |  | 5.73 | 3.949 x 10^-5^ |
|  |  |  | Low vs reference line | 15 |  | 6.33 | 1.348 x 10^-5^ |
|  |  |  | Intermediate vs reference line | 15 |  | 7.73 | 1.315 x 10^-6^ |
|  |  | Treatment |  | 2 | 1.15 |  | 0.326 |
|  |  | Residuals |  | 45 |  |  |  |
|  |  |  | 0 vs Low |  |  | 0.38 | 0.706 |
|  |  |  | 0 vs Intermediate |  |  | 1.46 | 0.151 |
| Tobramycin-Furanone C30 |  |  |  |  |  |  |  |
|  | - Atb |  | 0 vs reference line | 26 |  | -12.23 | 2.732 x 10^-12^ |
|  |  |  | Low vs reference line | 15 |  | -9.78 | 6.721 x 10^-8^ |
|  |  |  | Intermediate vs reference line | 15 |  | -4.55 | 3.844 x 10^-4^ |
|  | + Atb |  | 0 vs reference line | 26 |  | 8.80 | 2.815 x 10^-9^ |
|  |  |  | Low vs reference line | 26 |  | -3.17 | 0.004 |
|  |  |  | Intermediate vs reference line | 26 |  | -6.11 | 1.865 x 10^-6^ |
|  |  | Treatment |  | 2 | 71.61 |  | < 2.2 x 10^-16^ |
|  |  | Residuals |  | 78 |  |  |  |
|  |  |  | 0 vs Low |  |  | -9.26 | 3.390 x 10^-14^ |
|  |  |  | 0 vs Intermediate |  |  | -11.20 | < 2.0 x 10^-16^ |

^1^For each combination we performed a t-test. Degrees of freedom, t-values and p-values refer to the comparison to the zero line of the fitness of AtbR clones in presence of different concentrations of the antivirulence compound without the antibiotic.

^2^For each combination we performed an ANOVA. Degrees of freedom, F-values and p-values refer to the effect of the factor „Treatment“ on the fitness of AtbR clones.
