# Supplementary material for: Combining antibiotics with antivirulence compounds can have synergistic effects and reverse selection for antibiotic resistance in Pseudomonas aeruginosa
Source: PLoS Biol. 2020 Aug 18;18(8):e3000805. doi: 10.1371/journal.pbio.3000805 (PMC7433856; doi:10.1371/journal.pbio.3000805)
Supplement: S2 Table — (DOCX) [file pbio.3000805.s012.docx]

| **Compound** | **Media** | **Concentrations for combination experiments** | **Concentration for competitions** |
| --- | --- | --- | --- |
| Ciprofloxacin | CAA+Tf | 0.003, 0.006, 0.013, 0.025, 0.031, 0.05, 0.1, 0.25 (μg/ml) | 0.013 μg/ml |
|  | CAS | 0.03, 0.06, 0.13, 0.19, 0.25, 0.38, 0.5, 1 (μg/ml) | 0.25 μg/ml |
| Colistin | CAA+Tf | 0.005, 0.019, 0.023, 0.035, 0.053, 0.075, 0.15, 0.3 (μg/ml) | 0.075 μg/ml |
|  | CAS | 0.16, 0.31, 0.44, 0.63, 0.66, 0.99, 1.25, 1.48 (μg/ml) | 0.99 μg/ml |
| Meropenem | CAA+Tf | 0.03, 0.06, 0.125, 0.25, 0.5, 0.875, 1.75, 4 (μg/ml) | 0.25 μg/ml |
|  | CAS | 0.004, 0.008, 0.031, 0.063, 0.11, 0.22, 0.5, 1.75 (μg/ml) | 0.06 μg/ml |
| Tobramycin | CAA+Tf | 0.005, 0.019, 0.038, 0.075, 0.125, 0.15, 0.25, 0.6 (μg/ml) | 0.075 μg/ml |
|  | CAS | 0.063, 0.19, 0.5, 0.75, 1, 1.5, 3, 6 (μg/ml) | 0.75 μg/ml |
| Gallium | CAA+Tf | 0.47, 0.94, 1.56, 3.13, 6.25, 12.5, 25, 50 (μM) | 1.56 μM (low), 6.25 μM (intermediate) |
| Furanone C-30 | CAS | 3.05, 6.3, 22.8, 34.2, 51.4, 97.5, 195, 390 (μM) | 6.3 μM (low), 22.8 μM (intermediate) |
