# Supplementary material for: Combining antibiotics with antivirulence compounds can have synergistic effects and reverse selection for antibiotic resistance in Pseudomonas aeruginosa
Source: PLoS Biol. 2020 Aug 18;18(8):e3000805. doi: 10.1371/journal.pbio.3000805 (PMC7433856; doi:10.1371/journal.pbio.3000805)
Supplement: Attachment — Submitted filename: Rezzoaglie_etal_responses_to_comments.pdf [file pbio.3000805.s013.pdf]

COMMENTS FROM THE ACADEMIC EDITOR

Comments from the academic editor

I agree with the generally positive evaluation of the manuscript by the three reviewers. Thus I concur that the submitted manuscript is in principle suitable for PLoS Biol. I also agree that the manuscript should be further improved before it can be accepted. In particular, the suggestions from reviewer #1 for further experiments and data analyses should be considered (major points 1-3 from reviewer #1), as they would help to clarify some of the open points in the results. All three reviewers made helpful suggestions and comments as to how the text could be improved. These should be taken into account while revising the manuscript. I also agree with reviewer #3 that the clinical relevance of the data is not convincing. However, additional mouse experiments or clinical studies would go beyond the scope of the current study and the new insights obtained are already of sufficient importance as they may point to a new treatment strategy. Nevertheless, the authors should reconsider how they link their study to clinical reality and avoid any unrealistic claims that the current data does not support. The additional suggestions from reviewer #3 for further experiments and data analyses (see last three comments from reviewer #3) are very helpful and should be considered.

Response 1: We would like to thank the editor and the reviewers for their constructive comments. While we provide detailed responses to each of the comments below, we here wish to summarize that (i) we have conducted all the suggested extra in-vitro experiments; (ii) we have implemented the suggested edits, and have considered all the extra data analysis and discussion points proposed; and (iii) we agree that the clinical relevance of our work has to be put in the right context and that mouse experiments will be the next logic step to take in future studies.

Reviewer #1:

The aim of this study is to systematically assess the potential of novel combination treatments in which antibiotics (AB) are used together with antivirulence compounds (AV). AB+AV treatments are highly interesting as they may help to combat antibiotic resistance.

I enjoyed reading this piece of research. The experiments are straightforward and the manuscript is well written (clear and accessible to a wide audience). The authors focus on two AVs with distinct mechanisms (furanone, gallium) which are combined with four clinically relevant AB (CIP, MER, COL, TOB).

The authors claims are that

1) Certain AB+AV combinations produce clinically relevant levels of synergy
2) The interaction type (synergy, independent, antagonistic) is concentration dependent
3) AV can re-sensitize AB-resistant bacteria
4) AV+AB can select against resistance

These findings would be a significant contribution and suitable for publication with PLOS Biology. However, claims 3 + 4 are not sufficiently supported by the data, as important controls were missing (please see major point 1 below). I also have some technical concerns regarding the AV quantification (major point 2).

Response 2: thank you for this overall positive and constructive assessment. We agree that the additional experiments suggested below help to strengthen our conclusions and provide additional insights on antivirulence-antibiotic combination treatments. Following the reviewer’s advice, we have performed all the suggested experiments and report on them in detail below.

Major points
MP1. It is unclear whether the observed effects of re-sensitization and negative selection (Fig 5) are caused by drug interaction (synergy), or alternatively, and currently not considered by the authors, by the inhibitory effect of the AVs. This question can be addressed by experiments a, b.

a) Measure growth for treatments with AV alone (no antibiotic added) at the concentrations used in Fig 5A.

Response 3: We have carried out the suggested experiments and report the results in the main text on lines 221-248 and in the new Figure 5. Note that it was not possible to simply perform the suggested extra treatments and add them to the existing data sets, as all the treatments should be carried out at the same time. Accordingly, we have redone all the experiments with all the treatments from scratch. This was an enormous amount of work, but hugely paid off, because we could confirm the previous results, increase simple size, reduce variation and obtain additional conclusive insights. Specifically, we found that for gallium, the re-sensitization was because of the inhibitory effect of the antivirulence compound (new Figure 5A). For furanone, the patterns were more complex (new Figure 5B). For two antibiotics, there was no re-sensitization because of cross-resistance; for one antibiotic, re-sensitization occurred because of drug synergy; for another antibiotic, the growth inhibitory effect was established because of collateral sensitivity. Important to note is that we have removed one treatment in these sets of experiments, which is the combination treatment with the highest antivirulence compound concentration. This was done because this treatment was so potent that no bacteria could grow, which made it unsuitable for the questions addressed.

b) Competition experiments with AV alone (no antibiotics added) as an addition to Fig 5B. Antibiotic resistant strains frequently show hypersensitivity to other antibiotics (so-called collateral sensitivity). It is possible that similar phenomenon exists for AV, which may explain for example the negative selection of TOB-resistant bacteria in furanone-TOB combinations. This can be addressed by experiment c.

Response 4: We agree with the reviewer that these are important treatments. We have carried out the suggested experiments and present the results in the new Figure 6 and in the main text on lines 250-278. As with the experiment described above, we have redone all treatments simultaneously in many-fold replications. We could significantly decrease variation in the data and increase statistical power. The key findings of these experiments are: (i) When treated with antivirulence compounds alone, antibiotic resistant clones do not experience a selective advantage in 14 out of 16 tested cases. This demonstrates that the cost of antibiotic resistance is maintained when treated with antivirulence compounds. (ii) Our refined analysis now reveals five drug combos in which the addition of antivirulence compounds reduces, abrogates or reverses selection for antibiotic resistance. (iii) In the case of tobramycin-furanone, negative selection for resistance is indeed caused by collateral sensitivity.

c) AV dose-response analyses similar to Fig 2 for the resistant strains.

Response 5: This is a very valuable point. We have carried out the suggested experiments and present them in the new Supplementary Figure S5 together with statistical analysis. The new data indicate that antibiotic resistant clones can be both cross-resistant or collateral sensitive to antivirulence compounds, depending on the specific drug combination. The results reported here clearly support and help to explain the findings shown in the new Figures 5 and 6.

MP2. My understanding of virulence quenching is that the bacteria produce less AV per cell. However, it seems to me that the authors report the total amounts of virulence compound. As

evident from Fig 2 there is a strong positive correlation between total AV amount and cell density. It is therefore unclear whether the antivirulence quenching worked in the combinations (Figs 3 and 4). A possible solution would be to normalize the relative AV amount by relative growth.

Response 6: We agree that the theoretical view is that antivirulence compounds reduce the virulence factor production per cell. However, in reality the situation is more complex and depends on the specific regulatory circuits involved. For quorum-sensing controlled traits like proteases, the decrease in virulence factor production might not be gradual but follow a step-wise function. For pyoverdine, it is even more complicated as negative and positive feedbacks interact. We have previously shown that cells intermittently upregulate pyoverdine production at low gallium concentrations to compensate for the increased level of iron limitation, and down-regulate pyoverdine production at higher gallium concentrations because iron uptake via this pathway is unsuccessful (Ross-Gillespie et al. 2014 Evolution, Medicine, and Public Health). We recovered this finding with the data from this study (see graph below). We know explain these regulatory patterns in the introduction on lines 96-103.

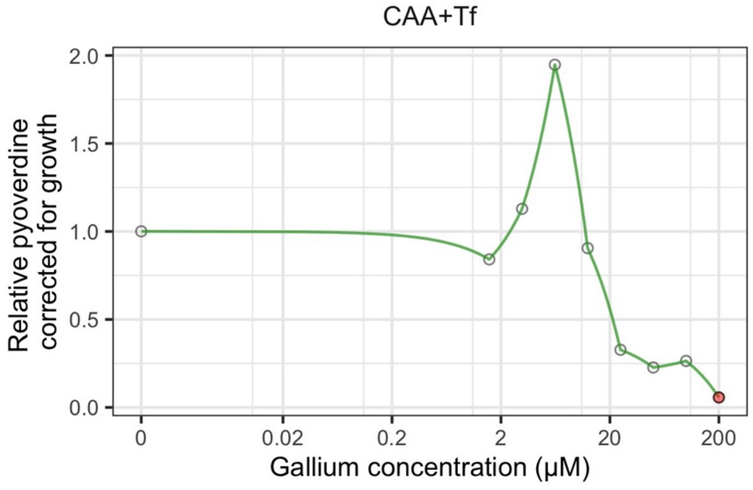

Naively, one would think that the upregulation of pyoverdine is bad for infections, but actually we could show that it is the other way round (Ross-Gillespie et al. 2014 Evolution, Medicine, and Public Health). The up-regulation of pyoverdine is a metabolic trap, where cells exhaust themselves in making pyoverdine that is subsequently rendered useless by gallium. These are complicated patterns, and because we have addressed them elsewhere, we do not wish to reiterate them in the current paper. Moreover, normalizing the data by growth is not possible because it would require a division by zero in treatments where there is no longer measurable growth.

Key is that gallium massively reduces pyoverdine availability at the population level. The same is true for protease quenching by furanone C-30 (Hentzer et al. 2002 Microbiology; 2003 EMBO Journal). The population-level reduction is the essential currency that determines treatment effects. Further note that in a medium where the virulence factor is essential, virulence factor quenching immediately feeds back on growth (now explained on lines 130-135). For example, in the iron-limited CAA medium used by us, the addition of gallium quenches pyoverdine, which induces iron starvation in cells and reduces population growth. This demonstrates that virulence factor quenching worked very well.

MP3. It is unclear whether cell density could affect the quantification of pyoverdine.

Response 7: We are not sure we fully understand what the reviewer means. Technically, the cell density does not affect the quantification of pyoverdine through excitation and fluorescence emission. We have done a lot of experiments with purified pyoverdine, where we could show that fluorescence increase linearly with quantity (see Schiessl et al. 2019 Evolution). We now clarify this on lines 504-505 in the methods.

But perhaps, this reviewer asks whether pyoverdine production is affected by population density. If so then the answer is yes. In this case, the regulatory mechanism we now introduce on lines 96-98 are involved.

The paper provides enough details of its methodology so that its experiments could be reproduced.
The authors treat the previous literature fairly.

Response 8: Thank you.

Minor points:
An explanation of why the observed effects are interpreted to provide "clinically interesting levels of synergy" (line 29) would be helpful.

Response 9: We have reconsidered this statement and we agree that at this stage, we have no basis to judge whether the observed levels of synergy are clinically interesting or not. The observation of synergy is simply promising. Thus, we have rephrased the sentence on line 32. We further discuss the clinical relevance of drug synergy in the discussion (349 onwards).

The words "is effective" in the title are unclear.

Response 10: This is a good point. Based on this comment, we have changed the title to "Combining antibiotics with antivirulence compounds can have synergistic effects and reverse selection for antibiotic resistance in *Pseudomonas aeruginosa*".

Line 318: for clarity please replace "induce" with "confer"

Response 11: We have changed the wording as per suggestion.

Please rationalize, why Bliss independence was used compared to Loewe additivity.

Response 12: We now provide justifications for using the Bliss independence model on lines 153+154 in the results and on lines 525-527 in the methods. Our rational for using this model is based on Baeder et al. 2015 Philos Trans R Soc B, who showed that the Bliss model is the adequate model for drugs with independent modes of actions, as it is the case in our study.

Reviewer #2:

To address the burning issue of antibiotic crisis, combination therapies are more and more recommended. Often, combination therapy is thought of as a combination of two antibiotics and the, mostly untested, rationale is that it is more difficult for bacteria two evolve two resistance mechanisms simultaneously. Here, the authors take a different approach. They investigate the efficacy of and the resistance evolution against a combination between antibiotics and adjuvants. Theses adjuvants are based on anti-virulence compounds. While, as the authors point out, such anti-virulence compounds have been investigated before also in combinations with antibiotics, the current manuscript clearly goes beyond that. It

systematically, across concentration gradients, evaluates interactions two-way interactions between two anti-virulence compounds in three relevant antibiotics and one relevant antimicrobial peptides (colistin). Importantly, this is combined with experimental evolution. The re-sequencing of the resultant strains also reveals that the mutations underlying the evolved resistant are those found from clinical strains.

Overall, this is a well-crafted manuscript with very interesting results. I feel that the manuscript would benefit from some clarifications and brief additional information in places:

Response 13: Thank you very much for the overall positive assessment.

Line 94 Please explain why you analyze symbiosis with Bliss independence and not for example with a Loewe approach. I have no issue with Bliss, it would just be good to know.

Response 14: We now provide justifications for using the Bliss independence model on lines 153+154 in the results and on lines 525-527 in the methods. Our rational for using this model is based on Baeder et al. 2015 Philos Trans R Soc B, who showed that the Bliss model is the adequate model for drugs with independent modes of actions, as it is the case in our study.

Line 101: Here, cross resistance is mentioned. But I cannot find data on cross-resistance. The authors infer the potential of cross-resistance from the mutations that result under experimental selection. This is fine, and I don't want to argue for additional experiments, but would suggest toning down cross-resistance here. Otherwise it would be useful to present data on cross-resistance of the selected lines.

Response 15: This reviewer is correct. Our evidence for cross-resistance was only indirect. Based on this and similar comments made by reviewer 1, we have conducted a series of extra experiments to address this issue. Specifically, we have established dose-response curve for all antibiotic resistant strains when exposed to the antivirulence compounds (see new Supplementary Figure S5 and new result section on lines 198-214). Moreover, we have redone all the mono-culture and competition assays with antivirulence compounds and antibiotics alone (new Figures 5+6). The new body of data reveals two clear cases of cross-resistance and one case of collateral sensitivity. See also our more detailed responses 3 and 4 above.

Line 176. There is not much detail given on the selection lines. It would be nice if the authors provided basic pharmacodynamic data in the supplement, such as MIC and perhaps also lag phase and growth rates (I assume they have got those data from their plate reader essays).

Response 16: To address this comment, we have established the dose-response curves for all antibiotic resistant clones (AtbR) and the wildtype for the four antibiotics used in the two different media (see lines 204-206 and new Supplementary Figure S4). Furthermore, we have estimated the half maximal inhibitory concentration (IC50) for both strains under all conditions, and compared them statistically. We found that AtbR clones had significant and many-fold increased IC50 values compared to the wildtype.

Line 224-250. It would be nice to provide a bit more information here. For example, on line 227 a singular is used and then it looks like more mutants are studied. Also, for Colistin 2 clones are mentioned, but more parallel lines are selected. Please make clear what the sample sizes are. Also, here background information is provided on the resistance mutations. While these resistance mutations fall within resistance mutations that have been described, they obviously do not cover the full spectrum that is known. Therefore it would be desirable in the discussion, when the authors hone in on particular combinations to make clear that this is also related to particular mutations and perhaps make a prediction on what would happen if other common mutations were involved.

Response 17: We are sorry that we were unclear about the sample size. Our aim was to analyze eight antibiotic resistant (AtbR) clones (one clone per antibiotic and media). In the end, we examined seven clones, as only one selection line survived the ciprofloxacin selection regime. We have now clarified this in the main text on lines 202-206 and explain our clone selection in detail in the methods on lines 560-571.

We further agree that our mutants arose through experimental evolution and only cover a certain spectrum of antibiotic resistance mechanisms. We elaborate on this aspect in more detail in the discussion (lines 439-443).

Line 269. I can see that the combinations tested here are potentially interesting. But has the toxicity of the anti-virulence compounds for patients been investigated. Could be briefly mentioned.

Response 18: This is a very valid point. Indeed, little thorough work is available on cyto-toxicity of these compounds. We now mention this and discuss the need for such tests on lines 437-439.

Lines 277-291. The point that the nature of interactions is concentration dependent is important. Perhaps it would be worth mentioning pharmacokinetics of drugs within patients which will determine the nature of interactions within patients.

Response 19: We have added a statement on this aspect in the discussion on lines 446-447.

Lines 311-312. I have already mentioned this above. The resistance mechanisms of the experimentally evolved strains make cross-resistance likely in some cases. It would have been desirable to test this, but as the paper is rich in data, I would suggest to at least discuss the issue of cross-resistance in more detail.

Response 20: As explained in our response 15 above, we have now conducted the required experiments, and provide evidence for cross-resistance in the new Supplementary Figure S5 and in the new Figure 5.

Fig. S1: I find it unusual to present correlations with a fitted regression line.

Response 21: We now clarify that the lines should be understood as trendlines for the associations. The use of trendlines is common for correlation plots. In fact, the Pearson's correlation coefficient r is the square root of the r^2 in the regression analysis, which means that our trendline is also mathematically correct.

Reviewer #3:

The authors assess growth inhibition and antibiotic resistance selection under multiple combinations of antibiotic and anti-virulence drugs, and report a fascinating mix of results. The most promising of these results are truly eye-catching: synergy (ie less total drug required for same effect) *AND* re-sensitization of AMR strains to antibiotics (ie AVs can be anti-resistance adjuvants) *AND* selection against antibiotic resistance. These are exciting claims and therefore require a high bar for acceptance in my view. Below are some of my concerns that I would want to see addressed –

Response 22: Thank you for this overall positive and constructive assessment. Below we address all the points raised by this reviewer in detail.

Address clinical relevance sooner and more seriously in the MS (this is the main issue). At present, there is a default sentence or two at the end on 'more work needed' towards clinical relevance. Given the motivation for this work is clinical, there needs to be better integration and earlier on. Let's take the best case, tobramycin + AVs, here the authors describe the striking combination of synergy, antibiotic repotentiation and reversal of resistance selection (WOW!). But the authors also report 'interaction hot-spots dependent on dose'. So the simple and addressable clinical relevance question becomes - do these exciting interactions happen under clinically relevant in vivo concentrations? This would require adding some in vivo benchmarks on the antibiotic doses (e.g. in vivo max; in vivo mean). These are upper thresholds - but it is also possible that these striking effects happen under relatively low dosing that (even with a synergy boost) are below reasonable clinical dosing goals. Both directions of clinical irrelevance should be directly assessed. I would also encourage the authors to consider taking their most promising results into a mouse model. They mention that in vivo would introduce spatial structure. That is just the start of it - in vivo would allow the virulence factors to actually have a virulence phenotype, plus impacts of immunity, pharmacodynamics, relevant nutritional environment, etc etc.

Response 23: We agree with the reviewer that the motivation of our work is clinical. In our revisions, we now mention this early on in the introduction (lines 67-74). Moreover, we mention that work on animal models are a next important step (lines 363+365). However, we wish to be careful (following the editor's suggestion) to not oversell our data. For the time being, our study consists of in-vitro work only, but we believe that, to our knowledge, there is no other study that has looked at antibiotic-antivirulence compounds interaction in such great detail and in the context of antibiotic resistance. The paper is already rich in data and we have discovered very promising drug combinations. We agree that the next step will be to test those in a mouse model. This is our plan, but we believe that this body of data (still to be collected) should be part of a follow-up paper.

Summarize a priori hypotheses at the front of the MS.
At present the MS is a bit of a laundry-list in places. Basically, the authors test a ton of interactions without stating clear testable hypotheses up front. This would help to sort the reams of data into results that are consistent with our current expectations and ones that are surprises (and therefore require careful attention).

Response 24: We are sorry that this reviewer was not happy with how we developed our storyline. We thought that we clarified early on that the aim of our study is to test whether antibiotic-antivirulence compound combinations could be potent drug treatments, because they combine two different approaches (killing and disarming). This then explains why we looked at drug interactions maps and how antibiotic resistant clones are affected by the drug combinations. We have now revised the introduction to further clarify these aspects (lines 67-74 and 112-116).

Resistance to AV?
Another path to treatment failure would be resistance to the AV component of a combination therapy. This is not assessed in this MS.

Response 25: We agree that resistance to antivirulence compounds is not part of this study. The reason is two-fold. First, resistance evolution to anti-virulence drugs (particularly to gallium and furanone) has been studied in detail by us and others. Second, we see antivirulence compounds as potentially potent adjuvants and that is why our primary focus was on antibiotic resistance. But we agree that antivirulence compound resistance evolution is an important point, and we now discuss this point and previous findings on lines 385-395 and 430-434.

Synergy significance threshold.
Lines 120-160 ish. Lots of statements on independence / non-independence, but no statistical support. What was the statistical test to support the claim that x-y was independent or synergistic under a given dosing, etc? multiple testing has to be considered.

Response 26: This is a good point. We have now conducted statistical tests on every single drug concentration combination and have corrected the p-values for multiple comparisons. We show the new analyses as p-value heat maps in the new Supplementary Figure S2. Many of the drug interactions, especially those at intermediate drug concentrations are indeed significant.

Re-sensitization
There's a sentence referencing the re-sensitization figure (fig 5A) that states that the AV doses have little direct effect on growth. This should be made clearer and quantified in the figure or figure legend. I attempted to gauge what direct effect the 'high' AV dose in fig 5A would have by referencing fig 2, and hard to get a clear figure due to log scale but could be 50% growth reduction which is substantial. The authors should spell out the direct effect here.

Response 27: We agree that the point of re-sensitization was not entirely clear and crucial control experiments were missing. Based on this comment and similar comments raised by reviewer 1, we have conducted a set of additional experiments, which provided conclusive results regarding re-sensitization (see our detailed responses 3+4 above).
