# Supplementary material for: Combining antibiotics with antivirulence compounds can have synergistic effects and reverse selection for antibiotic resistance in Pseudomonas aeruginosa
Source: PLoS Biol. 2020 Aug 18;18(8):e3000805. doi: 10.1371/journal.pbio.3000805 (PMC7433856; doi:10.1371/journal.pbio.3000805)
Supplement: Attachment — Submitted filename: Rezzoagli_PLOSBiol_R3_responses.docx [file pbio.3000805.s014.docx]

Editor

Thank you for submitting your revised Research Article entitled "Combining antibiotics with antivirulence compounds can have synergistic effects and reverse selection for antibiotic resistance in Pseudomonas aeruginosa " for publication in PLOS Biology. I have now obtained advice from the original reviewers and have discussed their comments with the Academic Editor.

As you can see, both reviewers feel the manuscript is significantly improved and have only minor comments. Rev. 3 does note however that his original request for clinical dose benchmarks has not been addressed. Based on the reviews, we will probably accept this manuscript for publication, assuming that you will modify the manuscript to address the remaining points raised by the reviewers. Please make sure to address the minor edits from Rev. 1 but also directly and explicitly discuss any limitations regarding clinical relevance. IMPORTANT: Please also make sure to address the data and other policy-related requests noted at the end of this email.

Response: Thank you for your positive assessment. We have addressed the final points raised by the reviewers and provide detailed responses below.

We expect to receive your revised manuscript within two weeks. Your revisions should address the specific points made by each reviewer. In addition to the remaining revisions and before we will be able to formally accept your manuscript and consider it "in press", we also need to ensure that your article conforms to our guidelines. A member of our team will be in touch shortly with a set of requests. As we can't proceed until these requirements are met, your swift response will help prevent delays to publication.

Response: We have received the formatting instructions and have worked through all the requests. Our manuscript should now meet the PLOS guidelines.

Reviewer #1:

I would like to congratulate the authors on a nice piece of research. I very much appreciate the additional experiments that were performed for the revisions. The new data now clarifies the previously open points, especially those regarding re-sensitization and selection inversion.

Altogether, I highly recommend the publication of this research with PLOS Biology.

Response: Many thanks for this positive assessment.

I do not have further scientific criticisms. Some very minor points that the authors may wish to edit:

1)	L. 91: I suggest to remove "antagonistically", as this could be confused with "antagonism"

Response: We have removed the word “antagonistically” from the respective sentence (now on line 88).

2)	L. 236: correct verb to "runs" or "ran"

Response: We have corrected the verb to “ran” (now on line 284).

3)	Fig 1: X-axis label has "Concentration" in caps, in contrast to the other figures

Response: We have changed the x-axis label of Fig. 1 to “Antibiotic concentration”.

---

Reviewer #3:

The authors have responded well to most of my earlier concerns, and I'm overall positive about this MS. However there's one important point that I mentioned in my first review that has not been addressed: clinical dose benchmarks.

I previously asked for clinical benchmarks, so that readers can begin to gauge whether the interesting phenomena are happening under conditions that are clinically relevant - ie between susceptible strain MICs and clinical break-points. This is a simple request and easy to address. For example for Tob, the question becomes, does the interesting behavior happen in the window of ~2 to 8 ug/ml? that's roughly the window between the MIC for wimpy PAO1 and a clinical breakpoint for resistance (sometimes higher). Unfortunately the PDF has been rendered by the journal with low resolution so I cant easily read the scale on fig 4 (not the authors fault), but looks like the synergy stops above zero-point-something ug/ml, ie below any relevant dose. If the interesting behavior for all drugs is outside of clinically relevant conditions, the paper is still interesting but the context and broader relevance does shift away from the clinic.

Response: Many thanks for your clarifications on this point, which we misunderstood during the first round of revisions. We agree that it would be ideal to directly compare the MIC and clinical breakpoints from clinical studies to the antibiotic concentration ranges used here. We now explicitly address this point in the discussion on lines 458-464. However, we wish to stress that a direct comparison is difficult, because absolute MIC- and breakpoint-values depend on the medium used and the specifics of the test setup (Turnidge and Paterson 2007 Clinical Microbiology Reviews 20: 391-408).

However, despite the fact that we used more growth-limiting media compared to the standard nutrient rich broth (because we required the bacteria to rely on the targeted virulence factors for growth), our inferred MICs (Fig. 1) are close to the average MIC-values (MIC50) reported across 7452 clinical strains *P. aeruginosa* (Sader et al. 2017 Antimicrobial Agents and Chemotherapy 61: e02252-16), see also Table below. We now mention this aspect on lines 458-464.

Moreover, important to note is that drug synergy can only be measured at antibiotic concentrations < MIC, because if antibiotics alone already completely stall bacterial growth then adding another drug has obviously no extra effect. This aspect can lead to the erroneous impression that we operate at a non-relevant concentration range. This is actually the key point of interactions therapy: to have two drugs at concentrations < MIC, and when combined they stall growth more than expected, ideally leading to complete growth inhibition.

**Table:** Comparison of MIC-values from Sader et al. 2017, our study and the clinical breakpoint values published by EUCAST (<https://www.eucast.org/clinical_breakpoints/>). All concentrations are in mg/l (=µg/ml). Values differ across media and studies.

MIC50 = Antibiotic concentration that inhibits 50% of the 7452 isolates

MIC90 = Antibiotic concentration that inhibits 90% of the 7452 isolates

MIC-CAA = MIC value measured in iron-limited casamino acids medium

MIC-CAS = MIC value measured in protease-dependent casein medium

S	= MIC for a susceptible strain

R	= Concentration above which a strain is considered resistant.
